# Consolidation radiotherapy for advanced-stage aggressive B-cell non-Hodgkin lymphoma: A systematic review and meta-analysis

**DOI:** 10.17179/excli2017-805

**Published:** 2017-11-21

**Authors:** Ernie Yap, Zhe Kang Law, Nik Muhd Aslan Abdullah, S. Fadilah Abdul Wahid

**Affiliations:** 1 Department of Medicine, UKM Medical Centre (UKMMC), University Kebangsaan Malaysia (UKM)., Jalan Yaakob Latif, 56000, Kuala Lumpur, Malaysia; 2 Department of Oncology, UKM Medical Centre (UKMMC), University Kebangsaan Malaysia (UKM)., Jalan Yaakob Latif, 56000, Kuala Lumpur, Malaysia; 3 Cell Therapy Centre, UKM Medical Centre (UKMMC), University Kebangsaan Malaysia (UKM)., Jalan Yaakob Latif, 56000, Kuala Lumpur, Malaysia

**Keywords:** non-Hodgkin lymphoma, radiotherapy, aggressive B cell NHL, meta-analysis

## Abstract

Patients with advanced aggressive B-cell non-Hodgkin lymphomas (NHL) are usually treated with rituximab in combination with chemotherapy. However, disease relapse rates are high. Radiotherapy (RT) has been shown to be efficacious in treating early-stage NHL but its role in advanced stage diseases is unclear. We performed a systematic review of randomized controlled trials (RCTs) comparing chemotherapy with RT to chemotherapy alone in patients with newly diagnosed advanced aggressive NHL. We searched online databases and pooled similar outcome estimates. For time-to-event outcomes, we estimated hazard ratios (HR) for overall survival (OS) and event-free survival (EFS) using the fixed-effect model. Two RCTs involving 254 patients met inclusion criteria. The trials were single-centre RCTs with follow-up period of five and ten years. Both trials were conducted in the pre-rituximab era. Patients treated with consolidation RT had better OS (HR for mortality 0.61; 95 % CI 0.38 to 0.97) and EFS (HR for mortality 0.67; 95 % CI 0.46 to 0.98) compared to those who received no RT. There was an apparent benefit of RT on local control (OR 0.09; 95 % CI 0.04 to 0.20); although this was estimated as a dichotomous rather than time-to-event outcome. Limited evidence shows benefits of consolidation RT in advanced aggressive NHL. However, we were not able to estimate the effect size with confidence due to small number of trials and sample size. We cannot recommend routine consolidation RT in advanced aggressive NHL. More RCTs with the inclusion of rituximab and PET-CT monitoring are needed.

## Introduction

Non-Hodgkin lymphomas are the most common lymphoid neoplasms of the lymphoid system in adults, accounting for about 4 % to 10 % of all malignancies worldwide (Müller et al., 2005[30]; Weisenburger, 1994[45]), of which diffuse large B-cell lymphoma (DLBCL) is the most common histological subtype (Harris et al., 1994[18], 1999[17]). The five-year survival rate for NHL is about 71 % for all patients (Siegel et al., 2014[40]). The 2008 World Health Organization classification (Swerdlow et al., 2008[43]) groups lymphomas by their corresponding cell of origin (B cell, T cell or NK cell) by employing immunophenotypic, cytogenetic and molecular methods. The mature B cell NHLs can be divided by their clinical behaviour into aggressive (fast-growing) and indolent (slow-growing), the former accounts for up to 50 % of all NHL patients.

Staging of the disease is performed according to the Ann Arbor staging model (Carbone et al., 1971[4]; Lister et al., 1989[24]).

Stage I and II are considered early stage whereas bulky stage II, stage III and IV are advanced stage. Early-stage diseases are usually treated with combination chemotherapy followed by radiation of affected sites and generally have good outcomes (Horning et al., 2004[22]; Miller et al., 1998[26])).

About 60 % to 70 % of people with aggressive NHL present in advanced stages of the disease (Swerdlow et al., 2008[43]). Advanced stage NHLs presents a challenging treatment paradigm to the treating clinician. Such patients require full dose rituximab-based chemotherapy to attain the best chance of a complete response (Fisher et al., 1993[14]). Although rituximab has significantly improved the prognosis of CD20+ B cell lymphomas, patients with advanced aggressive NHL still have high risk of disease relapse and death (Coiffier et al., 2010[6]; Schulz et al., 2007[36]).

Radiotherapy (RT) refers to the use of ionizing radiation to kill malignant cells. It is frequently employed in the treatment of bulky disease, which by itself is an independent adverse prognostic factor (Pfreundschuh et al., 2006[33]). RT given as consolidation therapy is effective in eradicating residual disease and improving local control. But whether this translates to a survival benefit is unclear. Routine addition of RT to areas of previously bulky diseases or residual masses in advanced NHL is not explicitly recommended in the National Comprehensive Cancer Network guidelines (Zelenetz et al., 2010[47]) which stipulates that one can consider the use of RT to initially bulky disease in selected cases. This is open to wide interpretation and the result is significant variation in clinical practice. As well, there is concern that the addition of RT may increase the risk of secondary malignancies, as extrapolated from patients with Hodgkin's lymphoma (Ng et al., 2002[31]; Dores et al., 2002[8]). Held et al. (2014[19]) performed a non-controlled prospective trial on the cohort with the best outcomes that emerged from a prior trial evaluating chemotherapy regimens (Pfreundschuh et al., 2008[32]) comparing consolidative RT with no further treatment. Event-free survival (EFS), progression-free survival (PFS) and overall survival (OS) were not significantly different but when only patients with bulky disease were analysed by intention-to-treat, superior EFS and a trend for better PFS and OS were seen in patients who received consolidation RT.

The optimum RT dose in the treatment of NHL is not as well studied compared to that for Hodgkin's lymphoma (Specht et al., 2014[41]). Most are in agreement that in the combined modality setting, a lower RT dose for control can be used, but minimum dose needed is not clear (Ferreri et al., 2000[13]; Kelsey et al., 2010[23]).

The addition of consolidation RT to bulky diseases has been shown to ameliorate the poor prognosis that bulky disease confers (Rübe et al., 2001[35]). Consolidation RT has been studied in *early-stage* aggressive NHL: Miller et al. (1998[26]) reported significant five-year PFS and OS benefit with the addition of consolidation RT after abbreviated chemotherapy compared with a prolonged course of chemotherapy alone. However, an updated report of the same study failed to show difference in failure-free survival and OS between the two treatment arms (Miller et al., 2011[27]). Bonnet et al. (2007[3]) found that the addition of 40 Gy of consolidation RT to initial affected sites did not improve survival in their cohort patients; all of whom were older than 60 years. Reyes et al. (2005[34]) compared intensive chemotherapy (ACVBP) alone with CHOP plus RT. With a median follow-up of 7.7 years, event-free and OS rates were significantly higher in the chemotherapy alone group.

The above mentioned studies have dampened enthusiasm for the use of consolidation RT in early-stage aggressive NHL although it can be argued that the lack of long term responses in the RT arm in the above trials were because RT was compared against abbreviated chemotherapy; as most mortality were attributed to disease relapse in distant sites. An Eastern Cooperative Oncology Group (ECOG) study that evaluated the question of consolidation RT in early aggressive NHL with a median follow-up of 12 years showed a 12 % gain in OS at six years with RT, although this was not statistically significant. The investigators also included a patient group who only achieved a PR after chemotherapy; all of whom received consolidation RT, and reported a six-year failure free survival rate of 63 % and OS rate of 69 % (Horning et al., 2004[22]). A subsequent systematic review and meta-analysis of RCTs that evaluated consolidative RT in localized aggressive NHL found no improvement in survival in adding RT to systemic chemotherapy; although significant heterogeneity across trials was noted (dos Santos et al., 2012[12]).

The role of RT in advanced aggressive NHL remains relevant in view of the markedly poorer prognosis that people with advanced disease have in comparison to their early-stage counterparts, highlighting the need for more intensive therapy. Shipp et al. (1989[39]) noted in their case series that bulky advanced-stage aggressive lymphomas usually relapsed in sites that were initially non-bulky or in new sites, arguing against the utility of consolidation RT. Nevertheless, the preponderance of retrospective observations seem to favor the use of RT (Ferreri et al., 2000[13]; Dorth et al., 2012[11][10]; Shi et al., 2013[38]) in this patient group. With the introduction of involved-field RT which spares the patient excessive doses of radiation while allowing treatment of difficult-to-access sites, this risk might have been mitigated. To the best of our knowledge, there is no systematic review with meta-analysis on this subject, although the authors of a narrative review recommended RT to bulky sites (Wirth, 2007[46]). 

## Methods

We included only randomized controlled trials (RCT)s which evaluated systemic (immuno)-chemotherapy with RT compared with systemic (immuno)-chemotherapy alone in people with advanced aggressive NHL. 

### Population

We included trials involving patients with newly diagnosed advanced-stage aggressive NHL who received systemic chemotherapy with or without rituximab with subsequent complete response (CR) or partial response (PR); of all ages, both sexes and all ethnicities. Aggressive NHL is defined by a clinical classification (Hiddemann et al., 1996[20]) and histological criteria. We accepted the following histological classifications: Working Formulation, Kiel-, REAL- and WHO-classification. The rationale for including patients with PR is based on the observation that people with radiological PR after chemotherapy may not have viable tumor within residual masses. As well, in trials where PR was shown to have predictive value, the management of such residual masses is not uniform (Surbone et al., 1988[42]; Dabaja et al., 2013[7]). Trials that include patients who did not achieve the above response rates were excluded. We also excluded patients with primary central nervous system lymphoma, primary gastrointestinal lymphoma, primary breast lymphoma, or primary testicular lymphoma.

### Interventions 

RT administered in the consolidation phase on patients with advanced, aggressive NHL who have achieved CR or PR from chemotherapy including alkylating agents, antimetabolites, topoisomerase inhibitors, anthracyclines, glucocorticoids or monoclonal antibody regimens. There had to be no restriction on dose, frequency, intensity or duration of RT.

### Comparator

Observation only in patients with NHL who have achieved CR or PR from (immuno)-chemotherapy including alkylating agents, antimetabolites, topoisomerase inhibitors, anthracyclines, glucocorticoids or monoclonal antibody regimens.

The (immuno)-chemotherapy regimens were to be the same in both arms, with equal cycles in both arms.

We excluded trials that include treatment in the disease-relapse setting.

### Primary outcome 

Overall survival (OS) defined as the time from entry into the clinical trial (random assignment in a phase III study) until death as a result of any cause (Cheson et al., 2007[5]).

### Secondary outcomes

- Event-free survival (EFS), defined as the interval from time of randomisation/study entry to an event (relapse, progression or death).
- Local control, defined as the absence of disease recurrence within the previously administered RT field in patients who received consolidation RT or at initially involved sites in both patients who did and did not receive RT, timed from the date of completion of chemotherapy, regardless of disease status outside of the field.
- Adverse events.

We searched following databases:

- MEDLINE (Ovid) (January 1946 to February 2017) (Appendix 1)
- EMBASE (1974 to February 2017) (Appendix 2)
- the Cochrane Central Register of Controlled Trials (CENTRAL) (*The Cochrane Library*, issue 4/2016) (Appendix 3)
- http://www.clinicaltrials.gov/.

### Data collection and analysis 

Two review authors (EY, ZKL) independently screened the abstracts of all studies identified for their eligibility for inclusion. If that was insufficient for a decision to be made, the full-text article was retrieved for a full review. Any disagreements were deferred and discussed with a third review author (SFAW). The number of studies identified, excluded and included was documented according to PRISMA (Moher et al., 2010[28])]. We used a standardized data extraction form.

### Assessment of risk of bias in included studies 

We assessed the risk of bias in the following domains: random sequence generation, allocation concealment, blinding of patients and personnel, blinding of outcome assessment, incomplete outcome data, selective outcome reporting and other potential sources of bias (Higgins and Green, 2011[21]).

### Measures of treatment effect 

For dichotomous outcomes, we used risk ratio (RR) and 95 % confidence interval (CI). For time-to-event data, we used HR and 95 % CI. If HR was not reported, we would have calculated it from the observed minus expected number of events (O-E) and variance for each end point. If these are not reported as well, we would have calculated HR with data extracted from Kaplan-Meier curves with the methods described by Tierney et al. (2007[44]). 

### Dealing with missing data 

We attempted to contact the authors of the primary studies to request missing data. We made explicit assumptions of any methods used. There were no substantial missing data in the included studies. Had there been substantial missing data, we would have imputed missing data for patients who were lost to follow-up after randomization (dichotomous data) assuming worse-case scenario for missing individuals. 

### Data synthesis 

We performed meta-analysis using the software Review Manager (RevMan) (*Version 5.3. Copenhagen: The Nordic Cochrane Centre, The Cochrane Collaboration, 2014)* and pooled HR for time-to-event outcomes using the O-E and variance method (HR < 1.0 is in favor of consolidative RT). O-E and variance were extracted based on number of events and p-value using methods described by Tierney et al. (2007[44]). We used a fixed-effect model for the primary analysis.

We assessed the quality of evidence according to each outcome using the Grading of Recommendations, Assessment, Development and Evaluations (GRADE) method which grade evidence into high, moderate, low and very low quality (Schünemann et al., 2013[37]).

## Results

### Results of the search 

**Figure 1:**
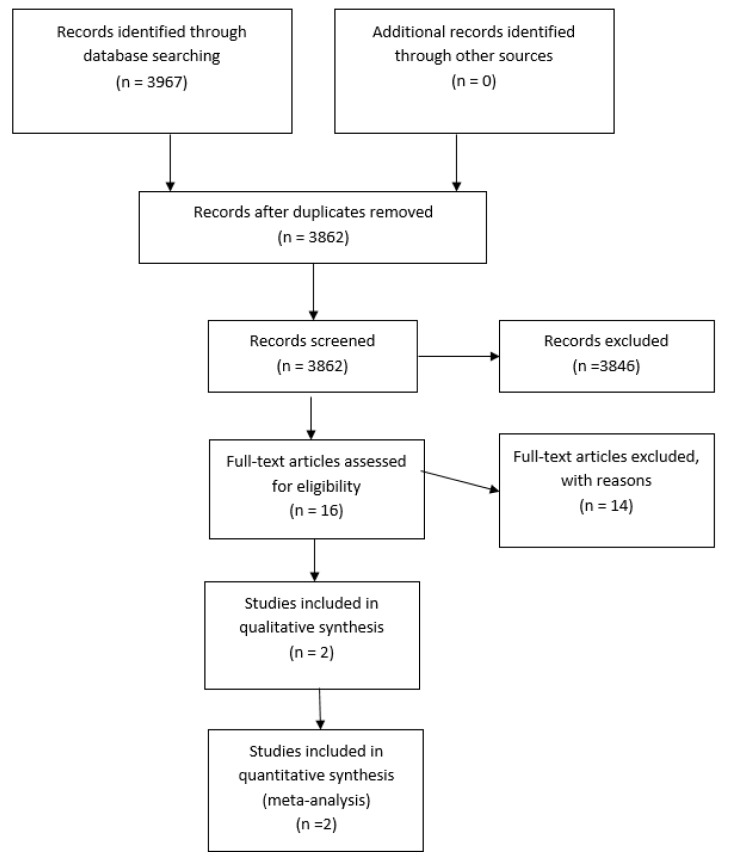
Flow diagram

Our search strategy resulted in 3862 possibly relevant articles from the databases cited above after de-duplication. Of these, 3846 were excluded at the initial screening stage as they did not fulfil the predefined inclusion criteria above. We extracted and evaluated 16 full-text publications. Of these, two studies were included in this systematic review. The screening, identification, selection, exclusion and inclusion of studies are documented according to the PRISMA flow diagram (Figure 1(Fig. 1)).

### Included studies 

Two studies were included: Avilés 1994 (Avilés et al., 1994[1]) and Avilés 2005 (Avilés et al., 2005[2])]. Both trials were performed at the National Medical Center, Mexico. In Avilés 1994[1], 88 patients with Stage IV diffuse large cell lymphoma in CR after systemic chemotherapy were randomized to receive RT to previous sites of bulky disease and no further treatment. The design of Avilés 2005[2] was slightly different in that 166 patients with stage III and IV diffuse large cell lymphoma (DLCL) who have residual disease (defined as nodal disease < 5 cm) were randomized to receive RT or observation.

### Patients 

Both trials included only patients with advanced DLCL (stage IV in Avilés 1994[1]; Stage III and IV in Avilés 2005[2]). Enrolment was between 1983 and 1997. Diagnosis was made according to the Working Formulation in Avilés 1994[1] and World Health Organization in Avilés 2005[2].

Avilés 1994[1] included only patients who achieved CR while Avilés 2005[2] included patients who had PR defined as residual mass of less than 5 cm after chemotherapy. People with residual extranodal disease and or had initial mass > 10 cm were not included.

All in all, 254 patients were enrolled between the years 1983 and 1997. Eighty-two patients were males. The median age ranged between 59 to 61.4 years.

### Interventions 

In Avilées 1994[1], all patients irrespective of treatment arm received initial chemotherapy that consisted of cyclophosphamide 600 mg/m^2^ intravenously (IV), vincristine 1.4 mg/m^2^ IV, epirubicin 70 mg/m^2^ IV on day 1; prednisone 50 mg/m^2^ orally on days 1-5, bleomycin 10 mg/m^2^ IV on day 14; with each cycle administered every 21 days x 2, followed by dexamethasone 20 mg/m^2 ^orally on days 1-5, cytosine arabinoside 1000 mg/m^2^ IV on days 2 and 3 and cisplatinum 100 mg/ m^2^ IV on day 1. The 88 enrolled patients who had achieved CR with initial bulky disease were randomised to one of two trial arms: RT to anatomic sites of previous bulky disease and no further treatment. Overall RT dose to upper abdomen was limited to 20 Gy in 20 fractions over 4 weeks. Kidneys were shielded posteriorly, reducing the dose to approximately 18 Gy. Regions of initial bulky disease were given additional boost of up to 25 Gy. Total doses to initial bulky sites were 45 Gy. Peripheral nodes and the upper mediastinum were treated with RT dose of 40 Gy in 20 fractions over 4 weeks whereas the dose of the lower mediastinum was approximately 25 % lower.

In Avilés 2005[2], all patients received 6 cycles of CHOP (cyclophosphamide, doxorubicin, vincristine, and prednisone) or CEOP (epirubicin 90 mg/m^2 ^instead of doxorubicin) prior to enrolment. RT was administered in doses of 30 Gy delivered in 20 doses, 1.5 Gy by fraction over four weeks on sites of residual nodal disease which is defined as presence of nodal mass < 5 cm.

### Comparator

The control arm of both Avilés 1994[1] and Avilés 2005[2] trials received standard chemotherapy as describe but not radiotherapy or sham radiotherapy.

### Outcomes 

Avilés 1994[1] reported primary outcome of OS and time to treatment failure (TTF). The authors also reported relapse at new and old sites as well as adverse events. Avilés 2005[2] used OS and progression-free disease (PFD) as study endpoints. The authors also reported number of relapses at initial residual sites, other anatomical sites and disseminated disease. Adverse events were reported albeit inadequately in both trials.

### Risk of bias in included studies 

Avilés 1994[1] did not describe methods used for generation of randomization but Avilés 2005[2] described randomization by blind envelope assigned by computer-designed numbered containers. Both trials were not blinded. Avilés 2005[2] reported intention-to-treat analysis. Both trials were published in peer-reviewed journals and reported ethics committee approval and informed consent by patients. Both trials did not utilise sham RT and hence performance bias was likely to be present. Measured outcomes such as OS and EFS were objective and unlikely to be biased in both trials. In Avilés 1994[1], patient withdrawal and lost to follow-up cases were not mentioned. As well, the investigators did not mention whether intention-to-treat or per-protocol analysis was done. In Avilés 2005[2], there was discordant data with regards to number of patients who died. Thus, we judged both trials to be of high risk in terms of attrition bias. In both trials, the study outcomes are routinely reported in standard hematology-oncological trials (survival, time to treatment failure and toxicity), thus it is unlikely that negative results were not reported.

### Effects of interventions 

#### OS

**Figure 2:**
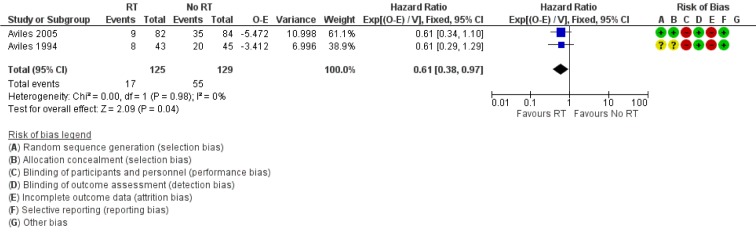
Forest plot of comparison: Overall survival. Events referred to death in each arm.

Both Avilés 1994[1] and Avilés 2005[2] were included in the analysis of OS. In Avilés 1994[1], 8 out of 43 patients in the RT arm died compared to 20 of 45 in the control arm. In Avilés 2005[2], 9 out of 82 patients in the RT arm died compared to 35 of 84 in the control arm. Patients treated with consolidation RT had a significantly better OS compared to no RT (HR for mortality 0.61 95 % CI: 0.38 to 0.97, Figure 2(Fig. 2); References in Figure 2: Avilés 1994[1], Avilés 2005[2]).

#### EFS

**Figure 3:**
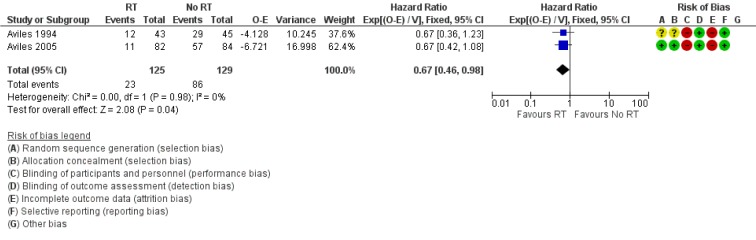
Forest plot of comparison: Event free survival. Events referred to relapse, progression or death in each arm.

Both Avilés 1994[1] and Avilés 2005[2] were included in the analysis of EFS. Although Avilés 1994[1] reported time to treatment failure and Avilés 2005[2] reported progression-free disease, both outcomes were synonymous with EFS. Avilés 1994[1] reported relapse in 12 patients in RT group compared to 29 in the control arm. In Avilés 2005[2], there were 11 patients who relapsed in the RT arm and all of them occurred outside the radiation field. In comparison, 57 patients had disease progression in the no-RT arm. Patients treated with consolidation RT had a significantly better EFS compared to no RT (HR for relapse, progression or death 0.67 95 % CI: 0.46 to 0.98, Figure 3(Fig. 3); References in Figure 3: Avilés 1994[1], Avilés 2005[2]).

### Local control

**Figure 4:**
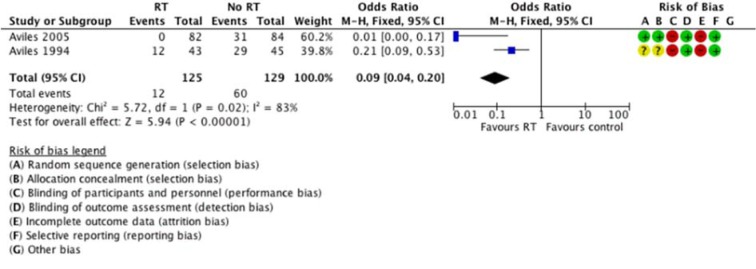
Forest plot of comparison: local control. Events referred to relapse at initial sites.

Neither study reported time-to-event data for local control; hence we reported local control as a dichotomous outcome, with relapse at initial disease site (old sites) as an event. Avilés 1994[1] reported relapse at old sites in 12 patients in RT arm and 29 patients in the control arm. Relapse involving new sites (non-RT treated sites) were reported in 4 patients in RT arm and 5 patients in control arm. 6 patients in RT arm and 9 patients in the control arm had relapses at both old and new sites. In Avilés 2005[2], none of the relapses in RT arm occurred within the RT field, while the control arm had 31 relapses at initial residual sites (old), 5 relapses at new sites and 23 had disseminated disease at time of relapse. The pooled estimate showed a significant benefit of radiotherapy on local control (Figure 4(Fig. 4); References in Figure 4: Avilés 1994[1], Avilés 2005[2]). However, this should be interpreted with caution; given the significant heterogeneity, small number of studies and patients. Also, estimate based on dichotomous outcome means we are uncertain of the duration local control was attained.

### Toxicity

Avilés 1994[1] reported grade 3 or 4 granulocytopenia in 41 out of 43 patients (95.3 %) in RT arm during chemotherapy resulting in 16 episodes of febrile illness, of which none were fatal. Eleven (25 %) of patients developed thrombocytopenia (platelet count < 100 X 10^9^/L). Only two patients with thrombocytopenia had bleeding which were considered minimal and did not require platelet transfusion. Thrombocytopenia was transient and all recovered between 7 to 19 days. Other adverse events included diarrhoea (n=2, 4.7 %) and elevated liver enzymes and bilirubin (n=1, 2.3 %). There was no cardiotoxicity observed up till one year after treatment. No secondary malignancies were observed with a median follow-up of 4.2 years. However, it was not clear if these were group-specific. 

Avilés 2005[2] only reported acute toxicity as mild and tolerable without mentioning the number of patients who suffered from toxicity in each arm. 2 patients from control arm had secondary malignancies while none from radiotherapy had them. We attempted unsuccessfully to reach the authors for clarification regarding toxicities in both trials.

### Quality of evidence

**Figure 5:**
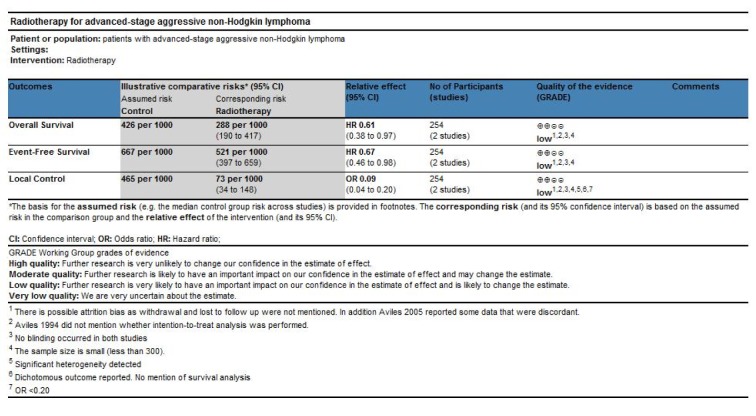
Summary of findings table

We graded the evidence as low quality for OS, EFS and local control. For OS and EFS, we downgraded one point for risk of bias and another point for imprecision. For local control, we downgraded one point each for risk of bias, imprecision, indirectness and inconsistency [see Figure 5(Fig. 5) (References in Figure 5: Avilés 1994[1], Avilés 2005[2]) and Discussion].

## Discussion

Our review demonstrated that RT has a modest benefit on OS, EFS and local control for people with advanced-stage aggressive non-Hodgkin lymphoma compared to observation. Due to incomplete reporting of adverse events, we were not able to perform meta-analysis on adverse effects of RT.

Our review has several limitations as both trials differ in their patient populations. Avilés 1994[1] randomized patients who have achieved CR after chemotherapy whereas Avilés 2005[2] randomized patients with residual disease (nodal mass < 5 cm) after chemotherapy. Despite those differences the results of both trials trended toward the same effect; that is favourably affecting EFS and OS. The benefit of RT on local control appeared to be greater in Avilés 2005[2]. This effect could be due to the trial recruiting patients with residual disease.

In addition, both trials were performed before rituximab became part of the standard of care. Due to rituximab's proven efficacy on disease outcomes, whether the addition of RT would provide additional benefits needs to be re-clarified in randomized controlled trials. Both trials also did not use positron emission tomography-computed tomography (PET-CT) scans that have an increasing role in guiding lymphoma treatment. PET-CT monitoring may help to select suitable candidates for RT and the extent of RT to be given. However, its use in predicting the efficacy of RT also has not been examined in RCTs.

Using the GRADE system of assessment, quality of evidence was graded as low for OS, EFS and local control. Both trials had high risk of bias in at least two components (blinding and attrition). Neither trial reported blinding of patients and personnel; including outcome assessors. This might introduce both performance bias (mainly for trials with an observation control arm) and detection bias for all trials. Only Avilés 2005[2] reported analysis on the basis of intention-to-treat. Number lost to follow up was not mentioned explicitly. Given the length of follow up for both trials, it is likely that some lost to follow up or withdrawal may have occurred. In addition, we accorded high risk for allocation concealment for Avilés 1994[1]. We also downgraded the score by one point for imprecision. The total number of patients were relatively small (<300). Despite using Cox regression for survival analysis, HR was not given in both trials. OS and EFS were reported as dichotomous outcome at specific timepoint (five year and ten years). We derived the HR from by calculating O-E and variance using number of events and p-values based on methods described by Tierney et al. (2007[44]). Local control was described as dichotomous outcome and survival analysis was not performed. Toxicities were reported as “mild and tolerable” in Avilés 2005[2] without mentioning type of toxicity specifically. Given these limitations, we are uncertain about the beneficial effect of RT on OS, EFS and local control.

Consolidation RT in bulky advanced aggressive NHL favourably affects PFS and OS according to existing data. However, more RCTs are needed in conjunction with personalized therapy approaches to evaluate this modality.

Much has been added into the armamentarium of NHL treatment since the above trials, yet RT remains a potent therapy. With the rising incidence of NHL and increasing life-expectancy of survivors, the role of consolidation RT needs to be re-evaluated in the rituximab era.

The optimum dose of RT remains to be defined. It has been demonstrated that radiation dose of more than 40 Gy provides better local control than less than 40 Gy (Fuller et al., 1995[15]) but this is somewhat intuitive and longer term effects need to be taken into account. Dorth et al. (2012[11][10]) retrospectively examined the efficacy of different RT doses in a single institution and found that patients in advanced stage were prone to receive lower doses of consolidation RT doses and there were no clear dose-response effects. The authors also found that the in-field failure was higher in sites more than or equal to 10 cm.

In a multicenter prospective randomized trial that compared differing RT doses in NHL, Lowry et al. (2011[25]) reported that in the aggressive NHL cohort of 640 patients, no significant difference was found between the group that received 30 Gy compared with 40-45 Gy in terms of freedom from progression, PFS and OS. The authors recommended 30 Gy as an efficacious standard dose for aggressive NHL.

The UNFOLDER trial (NCT00278408), was a RCT involving patients with disease > 7.5 cm randomized to 36 Gy involved field RT or no further treatment. The study was prematurely closed by the Independent Data Monitoring Committee due to a highly significant advantage in PFS in patients receiving RT (Mondello et al., 2015[29]). Data from that study will be instructive and lend further credence to the use of consolidative RT in advanced aggressive NHL.

Since the last analysed RCT Avilés 2005[2] was performed, much has evolved in terms of our understanding of NHL pathogenesis. Aside from histopathology, NHLs are further subtyped according to cell ontogeny; with the use of immunohistochemistry and gene expression profiling. Some NHL subtypes such as mantle cell lymphoma and primary mediastinal large cell lymphoma clearly have different biological behaviour and clinical prognosis and responsiveness to standard R-CHOP therapy. Indeed, new chemotherapy regimens are now recommended for these specific diseases. Although it would be unlikely that RT would produce different results in terms of local control, owing to the modality's non-specific nature; its impact on OS and EFS in the era of more guided chemotherapy would need to be evaluated prospectively; with longer follow-up periods. As well, diffuse large B cell lymphoma; the most common aggressive NHL is further subtyped according to gene expression profiles (Hans et al., 2004[16]). The germinal centre B cell (GCB) and activated B cell (ABC) subtypes each represents a biologically distinct tumor cell with GCB lymphomas were found to be more chemo-sensitive than the ABC subtype; thus conferring a better prognosis. Consolidative RT within this individualized treatment setting based on immunohistochemistry has not been explored.

An area of interest that warrants further study is the predictive value of PET-CT imaging in determining outcome and guiding additional therapy. In a retrospective study, a Duke University (Dorth et al., 2011[9]) a group analysed 99 DLBCL patients who received consolidation RT after positive post-chemotherapy PET or gallium scan and found approximately 65 % of them remained lymphoma-free after consolidation RT; although a vast majority of those analysed had early stage disease. 

Finally, in cases of relapsed advanced NHL, the current standard of care comprises of salvage chemotherapy followed by autologous stem cell transplant. The role of additional RT in this circumstance also requires further study.

## Acknowledgement

We would like to thank Professor Lai Nai Ming for his invaluable advice and guidance.

## Conflict of interest

The authors declare no conflict of interest.

## Appendices

**1 MEDLINE search strategy via Ovid**

1. exp LYMPHOMA, NON-HODGKIN/

2. (non-hodgkin* or non hodgkin* or nonhodgkin* or no hodgkin* or nhl).af.

3. (lymph* adj2 sarcom*).af.

4. lymphosarcom*.af.

5. (reticulum adj2 sarcom*).af.

6. (lymphom* adj2 (cleaved* or noncleaved* or grad* or mixed-cell* or pleomorphic*)).af.

7. (lymphom* adj2 (cleaved* or noncleaved* or grad* or mixed-cell* or pleomorphic* or diffus*)).af.

8. (bcell* or b-cell*).af.

9. or/1-8

10. *LYMPHOMA/

11. (lymphom* or linfom*).af.

12. exp HEMATOLOGIC NEOPLASMS/

13. (lympho* adj2 (neoplasm* or malign* or tumor* or tumour* or sarcom*)).af.

14. (lympha* adj2 (neoplasm* or malign* or tumor* or tumour* or sarcom*)).af.

15. (hemato* adj (malign* or neoplas*)).tw,kf,ot.

16. (haemato* adj (malign* or neoplas*)).tw,kf,ot.

17. or/10-16

18. 9 or 17

19. exp RADIOTHERAPY/

20. (radiotherap* or radio-therap*).tw,kf,ot.

21. exp LYMPHATIC IRRADIATION/

22. exp RADIOTHERAPY, IMAGE-GUIDED/

23. exp RADIOTHERAPY, COMPUTER-ASSISTED/

24. (radiotherap* or radiation* or irradiati*).tw,kf,ot.

25. or/19-24

26. 18 and 25

27. randomized controlled trial.pt.

28. controlled clinical trial.pt.

29. randomi?ed.ab.

30. placebo.ab.

31. clinical trials as topic.sh.

32. randomly.ab.

33. trial.ti.

34. or/27-33

35. humans.sh.

36. 34 and 35

37. 26 and 36

**2 EMBASE search strategy**

1. exp nonhodgkin lymphoma/

2. (non-hodgkin* or non hodgkin* or nonhodgkin* or no hodgkin* or nhl).tw.

3. (lymph* adj2 sarcom*).tw.

4. lymphosarcom*.tw.

5. (reticulum adj2 sarcom*).tw.

6. (lymphom* adj2 (cleaved* or noncleaved* or grad* or mixed-cell* or pleomorphic*)).tw.

7. (lymphom* adj2 (cleaved* or noncleaved* or grad* or mixed-cell* or pleomorphic* or diffus*)).tw.

8. (bcell* or b-cell*).tw.

9. or/1-8

10. *LYMPHOMA/

11. (lymphom* or linfom*).tw.

12. (lympho* adj2 (neoplasm* or malign* or tumor* or tumour* or sarcom*)).tw.

13. (lympha* adj2 (neoplasm* or malign* or tumor* or tumour* or sarcom*)).tw.

14. (hemato* adj (malign* or neoplas*)).tw.

15. (haemato* adj (malign* or neoplas*)).tw.

16. or/10-15

17. 9 or 16

18. exp RADIOTHERAPY/

19. (radiotherap* or radio-therap*).tw.

20. exp LYMPHATIC IRRADIATION/

21. exp IMAGE-GUIDED RADIOTHERAPY/

22. exp COMPUTER-ASSISTED RADIOTHERAPY/

23. (radiotherap* or radiation* or irradiati*).tw.

24. or/18-23

25. 17 and 24

26. (random$ or placebo$ or single blind$ or double blind$ or triple blind$).ti,ab.

27. RETRACTED ARTICLE/

28. or/26-27

29. (animal$ not human$).sh,hw.

30. (book or conference paper or editorial or letter or review).pt. not exp randomized controlled trial/

31. (random sampl$ or random digit$ or random effect$ or random survey or random regression).ti,ab. not exp randomized controlled trial/

32. 28 not (29 or 30 or 31)

33. 17 and 25 and 32

**3 CENTRAL search strategy**

1. MeSH descriptor: [Lymphoma] explode all trees

2. MeSH descriptor: [Hematologic Neoplasms] explode all trees

3. MeSH descriptor: [Hematologic Malignancies] explode all trees

4. (lymphom* or linfom*)

5. (non-hodgkin* or non hodgkin* or nonhodgkin* or no hodgkin* or non-hogkin* or non hogkin* or nonhogkin* or no hogkin* or non-hodkin* or non hodkin* or nonhodkin* or no hodkin* or non-hodgin* or non hodgin* or nonhodgin* or no hodgin* or nhl*)

6. (hemato* near/5 neoplas*) or (hemato* near/5 malign*) or (haemato* near/5 neoplasm*) or (haemato* near/5 malign*)

7. (bcell*) or (b*cell*)

8. #1 or #2 or #3 or #4 or #5 or #6 or #7

9. (lymph* adj2 sarcom*)

10. lymphosarcom*

11. (reticulum adj2 sarcom*)

12. (lymphom* adj2 (cleaved* or noncleaved* or grad* or mixed-cell* or pleomorphic* or diffus*))

13. (lympho* adj2 (neoplasm* or malign* or tumor* or tumour* or sarcom*))

14. (lympha* adj2 (neoplasm* or malign* or tumor* or tumour* or sarcom*))

15. #9 or #10 or #11 or #12 or #13 or #14

16. #8 or #15

17. MeSH descriptor: [Radiotherapy] explode all trees

18. MeSH descriptor: [Radiotherapy, Image-Guided] explode all trees

19. MeSH descriptor: [Radiotherapy, Computer-Assisted] explode all trees

20. MeSH descriptor: [Lymphatic Irradiation] explode all trees

21. (radiotherap* or radio-therap*)

22. (radiation therap*)

23. (Irradiati*)

24. #17 or #18 or #19 or #20 or #21 or #22 or #23

25. #16 and #24
